# Supplementary figures and images for: A bottom-up approach dramatically increases the predictability of body mass from personality traits
Source: PLoS One. 2024 Jan 10;19(1):e0295326. doi: 10.1371/journal.pone.0295326 (PMC10781087; doi:10.1371/journal.pone.0295326)

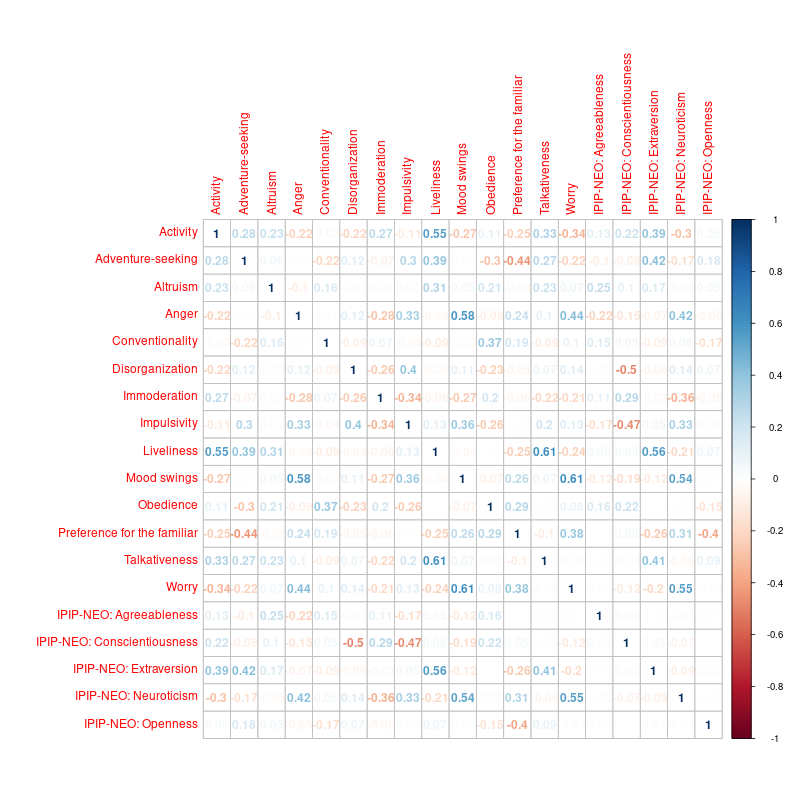

Supplement: S1 Fig — The factors and domains were calculated after residualizing the items for age, age2, sex, and continent. Factors and domains were calculated for people for whom at least three items in the factor (domain) were available. (PNG) [file pone.0295326.s007.png]
