# Supplementary material for: A bottom-up approach dramatically increases the predictability of body mass from personality traits
Source: PLoS One. 2024 Jan 10;19(1):e0295326. doi: 10.1371/journal.pone.0295326 (PMC10781087; doi:10.1371/journal.pone.0295326)
Supplement: Supplementary file 8 [file pone.0295326.s001.odt]

Table S1

Sample Characteristics

| Characteristic | Dataset |
| --- | --- |
| A | B | C |
| N | 43,972 | 37,870 | 19,716 |
| Age (years) | 26.33 (11.41) | 26.90 (11.29) | 26.36 (11.09) |
| Sex—n (%) |  |  |  |
| Male | 16,660 (37.89%) | 13,966 (36.88%) | 7,171 (36.37%) |
| Female | 27,312 (62.11%) | 23,904 (63.12%) | 12,545 (63.63%) |
| Education—n (%) |  |  |  |
| Less than 12 years | 6,364 (14.47%) | 5,997 (15.84%) | 2,570 (13.04%) |
| High school graduate | 3,564 (8.11%) | 3,235 (8.54%) | 1,791 (9.08%) |
| Currently in college/university | 18,402 (41.85%) | 14,605 (38.57%) | 8,043 (40.79%) |
| Some college/university, but did not graduate | 2,420 (5.50%) | 2,080 (5.49%) | 1,186 (6.02%) |
| College/university degree | 6,233 (14.17%) | 5,704 (15.06%) | 2,913 (14.77%) |
| Currently in graduate or professional school | 2,296 (5.22%) | 1,871 (4.94%) | 1,041 (5.28%) |
| Graduate or professional school degree | 3,934 (8.95%) | 3,629 (9.58%) | 1,880 (9.54%) |
| Continent—n (%) |  |  |  |
| Africa | 517 (1.18%) | 431 (1.14%) | 304 (1.54%) |
| Asia | 3,194 (7.26%) | 3,056 (8.07%) | 1,396 (7.08%) |
| Europe | 5,530 (12.58%) | 5,248 (13.86%) | 2,186 (11.09%) |
| North America | 32,993 (75.03%) | 27,748 (73.27%) | 15,051 (76.34%) |
| Oceania | 1,250 (2.84%) | 970 (2.56%) | 553 (2.80%) |
| South America | 488 (1.11%) | 417 (1.10%) | 226 (1.15%) |
| Body mass index (kg/m2) | 24.91 (5.84) | 25.19 (6.05) | 25.14 (5.99) |

Note. Means and SDs presented for age and body mass index.
