# Supplementary material for: A bottom-up approach dramatically increases the predictability of body mass from personality traits
Source: PLoS One. 2024 Jan 10;19(1):e0295326. doi: 10.1371/journal.pone.0295326 (PMC10781087; doi:10.1371/journal.pone.0295326)
Supplement: Supplementary file 9 [file pone.0295326.s002.odt]

S2 Table

Items Included in the Factors

| Item code | Item | Factor | Loading (A) | Loading (B) |
| --- | --- | --- | --- | --- |
| q_279 | Am usually active and full of energy. | Activity | .656 | .520 |
| q_1393 | Maintain high energy throughout the day. | Activity | .611 | .474 |
| q_1104 | Have great stamina. | Activity | .496 | .365 |
| q_832 | Feel healthy and vibrant most of the time. | Activity | .473 | .509 |
| q_595 | Do a lot in my spare time. | Activity | .367 | .316 |
| q_599 | Do dangerous things. | Adventure-seeking | .680 | .554 |
| q_1781 | Take risks. | Adventure-seeking | .548 | .578 |
| q_1662 | Seek adventure. | Adventure-seeking | .500 | .633 |
| q_2011 | Would love to explore strange places. | Adventure-seeking | .354 | .442 |
| q_1307 | Like to do things for others. | Altruism | .769 | .753 |
| q_1385 | Love to help others. | Altruism | .729 | .762 |
| q_1962 | Will do anything for others. | Altruism | .716 | .707 |
| q_1832 | Think of others first. | Altruism | .679 | .690 |
| q_1792 | Take time out for others. | Altruism | .616 | .609 |
| q_1053 | Have a soft heart. | Altruism | .572 | .535 |
| q_1162 | Inquire about others well-being. | Altruism | .547 | .556 |
| q_1159 | Immediately feel sad when hearing of an unhappy event. | Altruism | .367 | .377 |
| q_1364 | Love children. | Altruism | .343 | .417 |
| q_974 | Get irritated easily. | Anger | .776 | .662 |
| q_952 | Get angry easily. | Anger | .685 | .725 |
| q_101 | Am easily annoyed. | Anger | .676 | .576 |
| q_1357 | Lose my temper. | Anger | .631 | .742 |
| q_965 | Get easily agitated. | Anger | .567 | .586 |
| q_2036 | Yell at people. | Anger | .489 | .624 |
| q_995 | Get upset easily. | Anger | .425 | .433 |
| q_4300 | When my temper rises I find it difficult to control. | Anger | .393 | .512 |
| q_1653 | See myself as an average person. | Conventionality | .897 | .904 |
| q_525 | Consider myself an average person. | Conventionality | .867 | .870 |
| q_152 | Am just an ordinary person. | Conventionality | .823 | .779 |
| q_1300 | Like to be thought of as a normal kind of person. | Conventionality | .572 | .479 |
| q_747 | Enjoy being thought of as a normal mainstream person. | Conventionality | .448 | .440 |
| q_1254 | Leave a mess in my room. | Disorganization | .840 | .794 |
| q_1483 | Often forget to put things back in their proper place. | Disorganization | .663 | .627 |
| q_1255 | Leave my belongings around. | Disorganization | .649 | .665 |
| q_169 | Am not bothered by disorder. | Disorganization | .418 | .428 |
| q_1397 | Make a mess of things. | Disorganization | .384 | .438 |
| q_736 | Easily resist temptations. | Immoderation | .560 | .369 |
| q_56 | Am able to control my cravings. | Immoderation | .480 | .315 |
| q_1590 | Rarely overindulge. | Immoderation | .455 | .380 |
| q_1462 | Never splurge. | Immoderation | .430 | .386 |
| q_1461 | Never spend more than I can afford. | Immoderation | .389 | .324 |
| q_1120 | Have never engaged in gossip. | Immoderation | .322 | .410 |
| q_636 | Do things without thinking of the consequences. | Impulsivity | .557 | .650 |
| q_35 | Act without thinking. | Impulsivity | .556 | .616 |
| q_1424 | Make rash decisions. | Impulsivity | .504 | .556 |
| q_673 | Dont know why I do some of the things I do. | Impulsivity | .422 | .374 |
| q_4295 | Other people think of me as being very lively. | Liveliness | .504 | .552 |
| q_1803 | Talk to a lot of different people at parties. | Liveliness | .423 | .479 |
| q_4230 | Am rather lively. | Liveliness | .421 | .535 |
| q_1296 | Like to attract attention. | Liveliness | .395 | .380 |
| q_744 | Enjoy being part of a group. | Liveliness | .336 | .305 |
| q_497 | Change my mood a lot. | Mood swings | .799 | .776 |
| q_1099 | Have frequent mood swings. | Mood swings | .778 | .737 |
| q_4224 | My mood often goes up and down. | Mood swings | .759 | .860 |
| q_52 | Am a person whose moods go up and down easily. | Mood swings | .734 | .853 |
| q_979 | Get overwhelmed by emotions. | Mood swings | .404 | .362 |
| q_1752 | Stick to the rules. | Obedience | .685 | .614 |
| q_1867 | Try to follow the rules. | Obedience | .678 | .595 |
| q_4236 | People should always respect the law. | Obedience | .619 | .665 |
| q_369 | Believe laws should be strictly enforced. | Obedience | .593 | .651 |
| q_1657 | See that rules are observed. | Obedience | .568 | .496 |
| q_1624 | Respect authority. | Obedience | .564 | .531 |
| q_4291 | It is better to follow societys rules than to go my own way. | Obedience | .467 | .544 |
| q_1301 | Like to be viewed as proper and conventional. | Obedience | .371 | .386 |
| q_689 | Dont like the idea of change. | Preference for the familiar | .853 | .529 |
| q_566 | Dislike changes. | Preference for the familiar | .852 | .543 |
| q_1540 | Prefer to stick with things that I know. | Preference for the familiar | .485 | .396 |
| q_47 | Am a creature of habit. | Preference for the familiar | .415 | .483 |
| q_4226 | Am a talkative person. | Talkativeness | .829 | .836 |
| q_1795 | Talk a lot. | Talkativeness | .805 | .825 |
| q_1379 | Love to chat. | Talkativeness | .635 | .622 |
| q_4265 | Like telling jokes and funny stories to my friends. | Talkativeness | .464 | .425 |
| q_296 | Amuse my friends. | Talkativeness | .404 | .394 |
| q_1798 | Talk even when I know I shouldn’t. | Talkativeness | .393 | .455 |
| q_1244 | Laugh aloud. | Talkativeness | .354 | .413 |
| q_1064 | Have an intense boisterous laugh. | Talkativeness | .347 | .450 |
| q_1989 | Worry about things. | Worry | .793 | .646 |
| q_4256 | Worry about awful things that might happen. | Worry | .759 | .618 |
| q_4252 | Am a worrier. | Worry | .713 | .616 |
| q_808 | Fear for the worst. | Worry | .615 | .578 |
| q_986 | Get stressed out easily. | Worry | .461 | .366 |
| q_1505 | Panic easily. | Worry | .412 | .431 |

Note. Loadings are shown separately in datasets A and B.
