# Supplementary material for: A bottom-up approach dramatically increases the predictability of body mass from personality traits
Source: PLoS One. 2024 Jan 10;19(1):e0295326. doi: 10.1371/journal.pone.0295326 (PMC10781087; doi:10.1371/journal.pone.0295326)
Supplement: Supplementary file 10 [file pone.0295326.s003.odt]

S3 Table

Summary of Personality Variables’ Correlations With BMI

| Set of variables | Number of predictors | Correlations | Elastic net 1 |
| --- | --- | --- | --- |
| M | Range | Mdn | IQR | r | 95% CI |
| IPIP-NEO domains | 5 | .02 | .00–.04 | .01 | .01–.02 | .05 | .03–.07 |
| 14 factors | 14 | .09 | .03–.18 | .08 | .06–.10 | .15 | .13–.17 |
| Lead items | 14 | .08 | .03–.18 | .07 | .06–.09 | .15 | .13–.17 |
| IPIP-NEO facets | 30 | .02 | .00–.09 | .02 | .01–.03 | .05 | .03–.07 |
| Items in the 14 factors | 82 | .06 | .03–.18 | .05 | .04–.07 | .20 | .18–.22 |
| Items related to BMI left over | 160 | .05 | .02–.11 | .05 | .04–.06 | .19 | .17–.20 |
| All items related to BMI | 242 | .05 | .02–.18 | .05 | .04–.06 | .24 | .22–.26 |
| IPIP-NEO items | 298 | .03 | .00–.18 | .03 | .01–.04 | .19 | .17–.21 |
| All SAPA items | 694 | .03 | .00–.18 | .03 | .01–.04 | .24 | .22–.26 |

Note. Ranges and central tendencies of the correlations are based on absolute values. Correlations were calculated in the combined dataset. Factors and domains were calculated for people for whom at least 3 items in the respective factor or domain were available. Elastic net models were run in the subsample of participants for whom at least one domain, factor, and item in each set of variables was available (training data had 16,472 individuals and testing data, 9,565; missingness of personality data was 80% in each). IQR = interquartile range.

1 The p-value of all elastic net models was < .001 after applying false discovery rate correction.
