# Supplementary material for: A bottom-up approach dramatically increases the predictability of body mass from personality traits
Source: PLoS One. 2024 Jan 10;19(1):e0295326. doi: 10.1371/journal.pone.0295326 (PMC10781087; doi:10.1371/journal.pone.0295326)
Supplement: Supplementary file 11 [file pone.0295326.s004.odt]

S4 Table

Accuracy of Predicting BMI at Different Criteria of Interitem Correlations

| Criterion | Number of items | r 1 | 95% CI |
| --- | --- | --- | --- |
| 0.03 | 2 | .08 | .06–.10 |
| 0.05 | 3 | .09 | .07–.10 |
| 0.07 | 4 | .11 | .09–.13 |
| 0.10 | 7 | .12 | .10–.14 |
| 0.11 | 8 | .13 | .11–.15 |
| 0.12 | 12 | .14 | .12–.16 |
| 0.13 | 15 | .14 | .13–.16 |
| 0.14 | 17 | .15 | .13–.16 |
| 0.15 | 19 | .15 | .13–.17 |
| 0.16 | 20 | .15 | .13–.17 |
| 0.17 | 22 | .15 | .13–.17 |
| 0.18 | 24 | .15 | .13–.17 |
| 0.19 | 29 | .15 | .13–.17 |
| 0.20 | 30 | .15 | .13–.17 |

1 The p-value of all elastic net models was < .001 after applying false discovery rate correction.
