# Supplementary material for: A bottom-up approach dramatically increases the predictability of body mass from personality traits
Source: PLoS One. 2024 Jan 10;19(1):e0295326. doi: 10.1371/journal.pone.0295326 (PMC10781087; doi:10.1371/journal.pone.0295326)
Supplement: Supplementary file 12 [file pone.0295326.s005.odt]

S5 Table

The Predictive Scales

| Criterion | Item code | Item | Weight |
| --- | --- | --- | --- |
| .03 | q_56 | Am able to control my cravings. | -0.828 |
| .03 | q_1969 | Wish to stay young forever. | -0.029 |
| .05 | q_56 | Am able to control my cravings. | -0.846 |
| .05 | q_1969 | Wish to stay young forever. | -0.049 |
| .05 | q_2018 | Never go down rapids in a canoe. | 0.249 |
| .07 | q_56 | Am able to control my cravings. | -0.831 |
| .07 | q_1064 | Have an intense, boisterous laugh. | 0.409 |
| .07 | q_1969 | Wish to stay young forever. | -0.053 |
| .07 | q_2018 | Never go down rapids in a canoe. | 0.254 |
| .10 | q_56 | Am able to control my cravings. | -0.820 |
| .10 | q_711 | Don't strive for elegance in my appearance. | 0.423 |
| .10 | q_1064 | Have an intense, boisterous laugh. | 0.413 |
| .10 | q_1289 | Like music. | 0.134 |
| .10 | q_1969 | Wish to stay young forever. | -0.049 |
| .10 | q_2018 | Never go down rapids in a canoe. | 0.255 |
| .10 | q_4225 | Often take notice of what people think. | -0.038 |
| .11 | q_56 | Am able to control my cravings. | -0.802 |
| .11 | q_152 | Am just an ordinary person. | 0.392 |
| .11 | q_711 | Don't strive for elegance in my appearance. | 0.400 |
| .11 | q_1064 | Have an intense, boisterous laugh. | 0.411 |
| .11 | q_1289 | Like music. | 0.115 |
| .11 | q_1969 | Wish to stay young forever. | -0.033 |
| .11 | q_2018 | Never go down rapids in a canoe. | 0.233 |
| .11 | q_4225 | Often take notice of what people think. | -0.010 |
| .12 | q_56 | Am able to control my cravings. | -0.793 |
| .12 | q_152 | Am just an ordinary person. | 0.390 |
| .12 | q_397 | Believe that we coddle criminals too much. | 0.340 |
| .12 | q_463 | Can't do without the company of others. | -0.057 |
| .12 | q_711 | Don't strive for elegance in my appearance. | 0.410 |
| .12 | q_1064 | Have an intense, boisterous laugh. | 0.424 |
| .12 | q_1289 | Like music. | 0.111 |
| .12 | q_1691 | Seldom toot my own horn. | -0.087 |
| .12 | q_1969 | Wish to stay young forever. | -0.050 |
| .12 | q_2018 | Never go down rapids in a canoe. | 0.231 |
| .12 | q_4225 | Often take notice of what people think. | -0.023 |
| .12 | q_4263 | Worry about my health. | 0.319 |
| .13 | q_56 | Am able to control my cravings. | -0.776 |
| .13 | q_152 | Am just an ordinary person. | 0.388 |
| .13 | q_397 | Believe that we coddle criminals too much. | 0.347 |
| .13 | q_463 | Can't do without the company of others. | -0.064 |
| .13 | q_539 | Cry during movies. | 0.115 |
| .13 | q_711 | Don't strive for elegance in my appearance. | 0.410 |
| .13 | q_757 | Enjoy feeling "close to the earth." | -0.234 |
| .13 | q_1064 | Have an intense, boisterous laugh. | 0.426 |
| .13 | q_1289 | Like music. | 0.117 |
| .13 | q_1329 | Like to take it easy. | 0.439 |
| .13 | q_1691 | Seldom toot my own horn. | -0.096 |
| .13 | q_1969 | Wish to stay young forever. | -0.059 |
| .13 | q_2018 | Never go down rapids in a canoe. | 0.224 |
| .13 | q_4225 | Often take notice of what people think. | -0.035 |
| .13 | q_4263 | Worry about my health. | 0.331 |
| .14 | q_56 | Am able to control my cravings. | -0.767 |
| .14 | q_152 | Am just an ordinary person. | 0.389 |
| .14 | q_397 | Believe that we coddle criminals too much. | 0.339 |
| .14 | q_463 | Can't do without the company of others. | -0.067 |
| .14 | q_539 | Cry during movies. | 0.103 |
| .14 | q_711 | Don't strive for elegance in my appearance. | 0.406 |
| .14 | q_757 | Enjoy feeling "close to the earth." | -0.228 |
| .14 | q_1064 | Have an intense, boisterous laugh. | 0.417 |
| .14 | q_1289 | Like music. | 0.112 |
| .14 | q_1329 | Like to take it easy. | 0.450 |
| .14 | q_1373 | Love my enemies. | 0.160 |
| .14 | q_1691 | Seldom toot my own horn. | -0.088 |
| .14 | q_1969 | Wish to stay young forever. | -0.064 |
| .14 | q_2018 | Never go down rapids in a canoe. | 0.225 |
| .14 | q_2036 | Yell at people. | 0.314 |
| .14 | q_4225 | Often take notice of what people think. | -0.031 |
| .14 | q_4263 | Worry about my health. | 0.324 |
| .15 | q_56 | Am able to control my cravings. | -0.762 |
| .15 | q_152 | Am just an ordinary person. | 0.386 |
| .15 | q_397 | Believe that we coddle criminals too much. | 0.340 |
| .15 | q_463 | Can't do without the company of others. | -0.066 |
| .15 | q_539 | Cry during movies. | 0.102 |
| .15 | q_711 | Don't strive for elegance in my appearance. | 0.403 |
| .15 | q_757 | Enjoy feeling "close to the earth." | -0.229 |
| .15 | q_1064 | Have an intense, boisterous laugh. | 0.419 |
| .15 | q_1289 | Like music. | 0.117 |
| .15 | q_1329 | Like to take it easy. | 0.443 |
| .15 | q_1373 | Love my enemies. | 0.159 |
| .15 | q_1600 | React slowly. | 0.131 |
| .15 | q_1691 | Seldom toot my own horn. | -0.092 |
| .15 | q_1969 | Wish to stay young forever. | -0.068 |
| .15 | q_2018 | Never go down rapids in a canoe. | 0.218 |
| .15 | q_2036 | Yell at people. | 0.315 |
| .15 | q_4225 | Often take notice of what people think. | -0.038 |
| .15 | q_4263 | Worry about my health. | 0.319 |
| .15 | q_4269 | Suffer from sleeplessness. | 0.074 |
| .16 | q_56 | Am able to control my cravings. | -0.765 |
| .16 | q_152 | Am just an ordinary person. | 0.381 |
| .16 | q_397 | Believe that we coddle criminals too much. | 0.339 |
| .16 | q_463 | Can't do without the company of others. | -0.066 |
| .16 | q_539 | Cry during movies. | 0.102 |
| .16 | q_711 | Don't strive for elegance in my appearance. | 0.403 |
| .16 | q_757 | Enjoy feeling "close to the earth." | -0.225 |
| .16 | q_1064 | Have an intense, boisterous laugh. | 0.422 |
| .16 | q_1161 | Indulge in my fantasies. | -0.130 |
| .16 | q_1289 | Like music. | 0.122 |
| .16 | q_1329 | Like to take it easy. | 0.450 |
| .16 | q_1373 | Love my enemies. | 0.159 |
| .16 | q_1600 | React slowly. | 0.132 |
| .16 | q_1691 | Seldom toot my own horn. | -0.095 |
| .16 | q_1969 | Wish to stay young forever. | -0.064 |
| .16 | q_2018 | Never go down rapids in a canoe. | 0.217 |
| .16 | q_2036 | Yell at people. | 0.317 |
| .16 | q_4225 | Often take notice of what people think. | -0.039 |
| .16 | q_4263 | Worry about my health. | 0.320 |
| .16 | q_4269 | Suffer from sleeplessness. | 0.077 |
| .17 | q_43 | Always admit it when I make a mistake. | -0.123 |
| .17 | q_56 | Am able to control my cravings. | -0.757 |
| .17 | q_152 | Am just an ordinary person. | 0.382 |
| .17 | q_397 | Believe that we coddle criminals too much. | 0.340 |
| .17 | q_463 | Can't do without the company of others. | -0.067 |
| .17 | q_539 | Cry during movies. | 0.102 |
| .17 | q_711 | Don't strive for elegance in my appearance. | 0.403 |
| .17 | q_757 | Enjoy feeling "close to the earth." | -0.223 |
| .17 | q_1064 | Have an intense, boisterous laugh. | 0.423 |
| .17 | q_1120 | Have never engaged in gossip. | -0.055 |
| .17 | q_1161 | Indulge in my fantasies. | -0.131 |
| .17 | q_1289 | Like music. | 0.126 |
| .17 | q_1329 | Like to take it easy. | 0.448 |
| .17 | q_1373 | Love my enemies. | 0.165 |
| .17 | q_1600 | React slowly. | 0.132 |
| .17 | q_1691 | Seldom toot my own horn. | -0.091 |
| .17 | q_1969 | Wish to stay young forever. | -0.069 |
| .17 | q_2018 | Never go down rapids in a canoe. | 0.217 |
| .17 | q_2036 | Yell at people. | 0.308 |
| .17 | q_4225 | Often take notice of what people think. | -0.039 |
| .17 | q_4263 | Worry about my health. | 0.319 |
| .17 | q_4269 | Suffer from sleeplessness. | 0.076 |
| .18 | q_43 | Always admit it when I make a mistake. | -0.109 |
| .18 | q_56 | Am able to control my cravings. | -0.752 |
| .18 | q_152 | Am just an ordinary person. | 0.362 |
| .18 | q_397 | Believe that we coddle criminals too much. | 0.325 |
| .18 | q_403 | Bottle up my feelings. | 0.281 |
| .18 | q_463 | Can't do without the company of others. | -0.055 |
| .18 | q_539 | Cry during movies. | 0.100 |
| .18 | q_711 | Don't strive for elegance in my appearance. | 0.401 |
| .18 | q_757 | Enjoy feeling "close to the earth." | -0.215 |
| .18 | q_1064 | Have an intense, boisterous laugh. | 0.424 |
| .18 | q_1120 | Have never engaged in gossip. | -0.055 |
| .18 | q_1161 | Indulge in my fantasies. | -0.115 |
| .18 | q_1289 | Like music. | 0.134 |
| .18 | q_1329 | Like to take it easy. | 0.438 |
| .18 | q_1373 | Love my enemies. | 0.174 |
| .18 | q_1600 | React slowly. | 0.120 |
| .18 | q_1691 | Seldom toot my own horn. | -0.101 |
| .18 | q_1861 | Try to avoid complex people. | 0.368 |
| .18 | q_1969 | Wish to stay young forever. | -0.080 |
| .18 | q_2018 | Never go down rapids in a canoe. | 0.202 |
| .18 | q_2036 | Yell at people. | 0.298 |
| .18 | q_4225 | Often take notice of what people think. | -0.039 |
| .18 | q_4263 | Worry about my health. | 0.308 |
| .18 | q_4269 | Suffer from sleeplessness. | 0.064 |
| .19 | q_43 | Always admit it when I make a mistake. | -0.093 |
| .19 | q_56 | Am able to control my cravings. | -0.732 |
| .19 | q_152 | Am just an ordinary person. | 0.347 |
| .19 | q_251 | Am seldom bothered ... suffering of strangers. | 0.059 |
| .19 | q_397 | Believe that we coddle criminals too much. | 0.305 |
| .19 | q_403 | Bottle up my feelings. | 0.260 |
| .19 | q_463 | Can't do without the company of others. | -0.043 |
| .19 | q_539 | Cry during movies. | 0.089 |
| .19 | q_711 | Don't strive for elegance in my appearance. | 0.385 |
| .19 | q_757 | Enjoy feeling "close to the earth." | -0.191 |
| .19 | q_1064 | Have an intense, boisterous laugh. | 0.417 |
| .19 | q_1120 | Have never engaged in gossip. | -0.037 |
| .19 | q_1161 | Indulge in my fantasies. | -0.093 |
| .19 | q_1289 | Like music. | 0.118 |
| .19 | q_1329 | Like to take it easy. | 0.416 |
| .19 | q_1373 | Love my enemies. | 0.163 |
| .19 | q_1553 | Put little time and effort into my work. | 0.088 |
| .19 | q_1600 | React slowly. | 0.095 |
| .19 | q_1691 | Seldom toot my own horn. | -0.084 |
| .19 | q_1861 | Try to avoid complex people. | 0.346 |
| .19 | q_1969 | Wish to stay young forever. | -0.068 |
| .19 | q_2018 | Never go down rapids in a canoe. | 0.185 |
| .19 | q_2036 | Yell at people. | 0.281 |
| .19 | q_4222 | Have many different hobbies. | -0.145 |
| .19 | q_4225 | Often take notice of what people think. | -0.020 |
| .19 | q_4227 | Being in debt is worrisome to me. | 0.013 |
| .19 | q_4233 | Dislike people who don't know how to behave themselves. | 0.033 |
| .19 | q_4263 | Worry about my health. | 0.296 |
| .19 | q_4269 | Suffer from sleeplessness. | 0.052 |
| .20 | q_43 | Always admit it when I make a mistake. | -0.090 |
| .20 | q_56 | Am able to control my cravings. | -0.730 |
| .20 | q_152 | Am just an ordinary person. | 0.346 |
| .20 | q_251 | Am seldom bothered ... suffering of strangers. | 0.058 |
| .20 | q_397 | Believe that we coddle criminals too much. | 0.303 |
| .20 | q_403 | Bottle up my feelings. | 0.258 |
| .20 | q_463 | Can't do without the company of others. | -0.042 |
| .20 | q_539 | Cry during movies. | 0.087 |
| .20 | q_711 | Don't strive for elegance in my appearance. | 0.383 |
| .20 | q_757 | Enjoy feeling "close to the earth." | -0.190 |
| .20 | q_1064 | Have an intense, boisterous laugh. | 0.415 |
| .20 | q_1120 | Have never engaged in gossip. | -0.035 |
| .20 | q_1161 | Indulge in my fantasies. | -0.092 |
| .20 | q_1289 | Like music. | 0.114 |
| .20 | q_1329 | Like to take it easy. | 0.414 |
| .20 | q_1373 | Love my enemies. | 0.162 |
| .20 | q_1553 | Put little time and effort into my work. | 0.087 |
| .20 | q_1600 | React slowly. | 0.093 |
| .20 | q_1691 | Seldom toot my own horn. | -0.083 |
| .20 | q_1861 | Try to avoid complex people. | 0.344 |
| .20 | q_1969 | Wish to stay young forever. | -0.066 |
| .20 | q_2018 | Never go down rapids in a canoe. | 0.183 |
| .20 | q_2036 | Yell at people. | 0.279 |
| .20 | q_4222 | Have many different hobbies. | -0.144 |
| .20 | q_4225 | Often take notice of what people think. | -0.019 |
| .20 | q_4227 | Being in debt is worrisome to me. | 0.011 |
| .20 | q_4233 | Dislike people who don't know how to behave themselves. | 0.030 |
| .20 | q_4263 | Worry about my health. | 0.294 |
| .20 | q_4269 | Suffer from sleeplessness. | 0.050 |
| .20 | q_4298 | Am touchy about some things. | 0.023 |
