# Supplementary material for: A bottom-up approach dramatically increases the predictability of body mass from personality traits
Source: PLoS One. 2024 Jan 10;19(1):e0295326. doi: 10.1371/journal.pone.0295326 (PMC10781087; doi:10.1371/journal.pone.0295326)
Supplement: Supplementary file 13 [file pone.0295326.s006.odt]

S6 Table

Observed BMI and Weight by Predicted BMI Decile

|  | 4 items | 17 items |
| --- | --- | --- |
| Decile | BMI (kg/m2) | Weight (kg) | BMI (kg/m2) | Weight (kg) |
| 1 | 24.32 | 68.54 | 23.71 | 67.74 |
| 2 | 25.12 | 73.52 | 24.49 | 70.39 |
| 3 | 25.22 | 72.58 | 24.59 | 70.74 |
| 4 | 25.09 | 72.22 | 24.84 | 71.92 |
| 5 | 24.87 | 71.23 | 24.95 | 71.23 |
| 6 | 24.94 | 72.13 | 24.76 | 71.20 |
| 7 | 24.93 | 71.71 | 24.91 | 71.90 |
| 8 | 24.72 | 71.04 | 25.01 | 72.03 |
| 9 | 24.81 | 71.22 | 25.87 | 74.53 |
| 10 | 25.72 | 73.61 | 26.61 | 76.14 |
